# A Non-operative Approach of Small Bowel Obstruction in Virgin Abdomens

**DOI:** 10.7759/cureus.57206

**Published:** 2024-03-29

**Authors:** Bhovineey Ramanathan, Vinod Ramachandran, Abdul Rana, Christopher McDonald

**Affiliations:** 1 Department of Surgery, Lyell McEwin Hospital, Elizabeth Vale, AUS; 2 Department of Colorectal Surgery, Northern Adelaide Local Health Network, Adelaide, AUS

**Keywords:** sbo-va, surgical approach, conservative, virgin abdomen, small bowel obstruction

## Abstract

We present a compelling case of small bowel obstruction (SBO) in a 38-year-old male with a virgin abdomen, a term used to describe an individual who has not undergone prior abdominal surgery. Despite his fit and healthy status, he presented with symptoms indicative of bowel obstruction. Through a meticulous series of history-taking, comprehensive clinical examinations, and precise imaging studies, we were able to arrive at a conclusive diagnosis. Remarkably, the patient experienced a full recovery solely through conservative management, effectively sidestepping the need for surgical intervention. This case prompts a deeper discussion on the nuanced approaches to SBO in individuals with virgin abdomens. We aim to delve into the comparative merits of conservative versus surgical strategies, considering the latest evidence-based practices to guide our understanding and decision-making in such cases.

## Introduction

Virgin abdomens are defined as abdomens with no previous surgeries, peritoneal adhesions or diseases, or radiotherapy. The standard practice was the investment of early surgical intervention for patients who have developed small bowel obstruction in the virgin abdomen (SBO-VA) [1]. This is in the background thought of contemplating a possible malignant aetiology or a more complicated/catastrophic aetiology compared to someone who has undergone previous abdominal operations. However, the latest research [2] has seemed to shift from this dogma. Unfortunately, up to date, no concrete study is present to confirm that foregoing operative management in patients with no prior operative history is not harmful.

## Case presentation

The patient in our study is a 38-year-old male with a virgin abdomen who presented with his first episode of SBO. He initially presented to the Emergency Department within six hours of the onset of generalised abdominal pain and a few episodes of vomiting. Apart from being a smoker, he is otherwise a fit and healthy guy. Vitals on arrival were all normal and physical examination revealed a generalised tender abdomen with guarding but no signs of peritonitis. Bowel sounds were faint. Initial laboratory investigations revealed no abnormalities.

The differential diagnosis we were considering at this point for a young and healthy male who presented with an acute abdomen of sudden onset was SBO, appendicitis, diverticulitis, Meckel's diverticulum, gastroenteritis, inflammatory bowel diseases, possible perforated viscus, pyelonephritis and epiploic appendagitis. Other less pertinent causes like muscular strain were still a consideration but were kept lower on the list.

**Figure 1:**
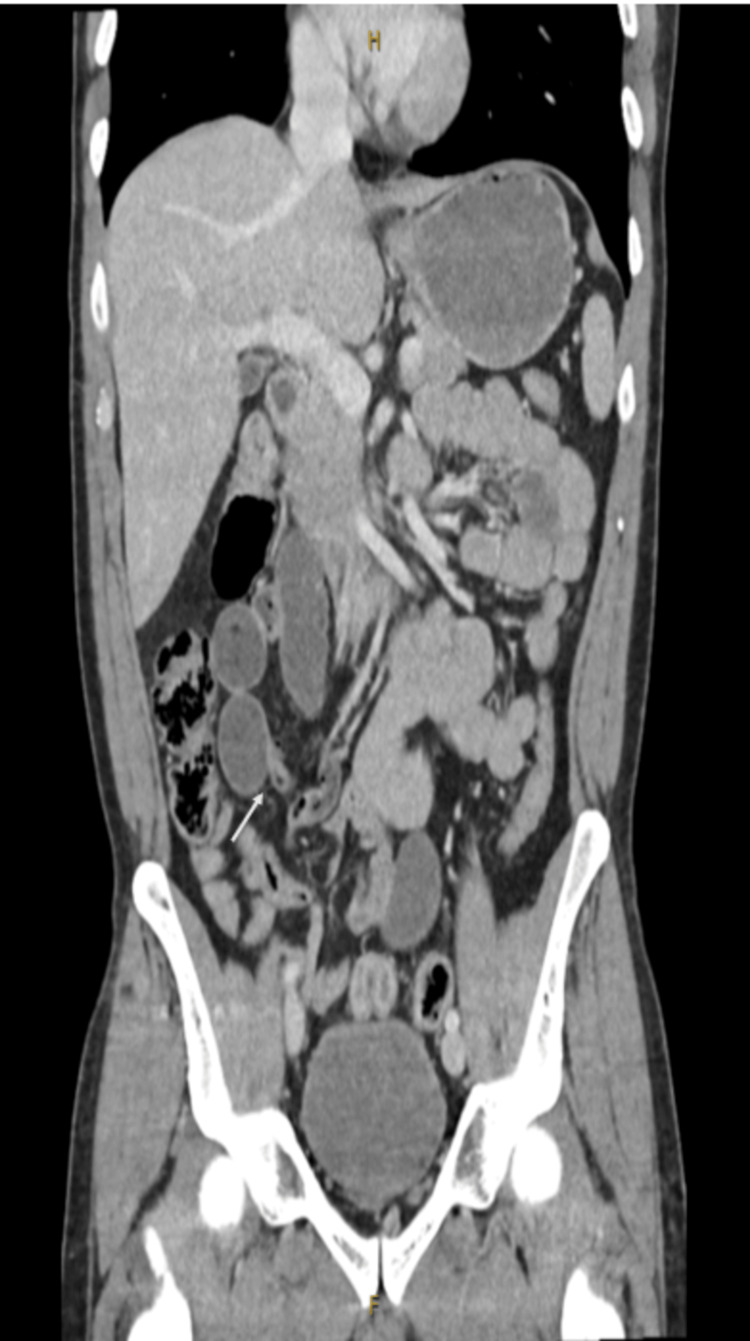
Coronal view of the CT scan revealing small bowel loops measuring up to 3 cm and collapsed distal small bowel. The arrow pointing towards the transition point.

As the baseline blood tests did not provide the necessary information, a computed tomography (CT) scan with IV contrast was performed as the patient’s pain was not improving even with analgesics. The CT scan (Figure 1) revealed groups of distended small bowel loops seen on the right side of the abdomen measuring up to 3 cm in diameter. The distal small bowel is collapsed. The proximal small bowel is of normal calibre. The stomach is slightly distended with fluid. The finding raises the suspicion of SBO or closed-loop obstruction. Most of the large bowels collapsed. No free fluid or collection was seen in the abdomen or pelvis.

Upon having a CT confirmed SBO, a 16 gauge nasogastric tube was immediately inserted to decompress the stomach which provided much relief. A urinary catheter was also inserted to monitor the hydration status of the patient. Since this patient was seen in a smaller hospital with limited surgical capabilities and no emergency theatre capacity, an immediate transfer was arranged to the nearest tertiary hospital after a proper handover was given to the surgical doctor on the other side. However, fortunately, the patient’s pain was much better when he arrived at the tertiary hospital to the extent he was clinically examined as having a soft and non-tender abdomen. He was managed conservatively (with just nasogastric decompression, IV fluid resuscitation and analgesics) and was able to pass flatus the next day without even a trial of Gastrografin® and was subsequently discharged the following day upon gradual upgrade of diet. The patient has been followed up for six months after the occurrence of the first SBO and he has never had a recurrence of SBO to date.

## Discussion

There have been a few studies in the past that investigated the occurrence of SBO-VA. There are many causes of SBO-VA as per the literature search that has been done. Among them include adhesions (being the most common), internal herniation, intussusception, gallstone ileus, phytobezoar, newly diagnosed small bowel tumour, stricture, Meckel's diverticulum and mesenteric volvulus [1]. Similar to our patient in this report, the majority of SBO-VA happens to be happening in the male population (66.7%) [2] compared to the female population. The median age [2] in years of occurrence happens to be 58 years but the range is between 23 and 101.

According to systemic reviews and meta-analysis done on this topic [3], it has been noted that successful non-operative SBO management in patients without prior abdominopelvic surgery might actually risk missing a potential malignant cause. However, this does not warrant surgery for all SBO-VA even if they are clinically not more obstructed. But this means that further workup is warranted for patients with SBO-VA even upon discharge as this might reduce the chances of us missing a malignant or catastrophic cause.

CT can be used to diagnose some SBO aetiologies such as foreign body obstruction, gallstone ileus and phytobezoar [3]. Occasionally, diagnostic laparoscopy may even be considered for patients for whom a malignant aetiology cannot be ruled out. If non-operative management is the choice of managing a particular patient, subsequent outpatient follow-up and diagnostic work-up seem to be appropriate for certain low-risk patients (those with no previous bowel issues, comorbidities, generally healthy otherwise).

The main question of whether SBO-VA can be treated with the same approach as an SBO in someone who had previous surgeries in the past is yet to be answered. There are many conflicting data available on this topic. I would like to use a reference from a WSES (World Society of Emergency Surgeons) position paper to explain this further. A group of doctors [4] conducted a narrative review with scoping aspects involving seven original studies in this subject matter. The conclusion from the study was that since the aetiology and treatment results for patients with SBO-VA are largely comparable with those with previous surgeries, hence they could be treated according to the existing guidelines for SBO and adhesive SBO.

Gastrografin also known as diatrizoate [5] uses an iodinated, hyperosmotic, radiopaque contrast medium that leads to the resolution of adhesive SBO occasionally. There is not much research available on the success of the use of Gastrografin as a treatment for SBO-VA but since the latest consensus was to treat SBO-VA in a similar way as adhesive SBO, hence the use of Gastrografin seems to be justified. A case report is available on the success of Gastrografin in treating SBO-VA [6].

A retrospective study [7] highlights that the rate of recurrence in patients with SBO-VA managed conservatively was only 7.9%; however, this was a small study that only involved 63 patients with virgin abdomens. The data suggest that selected patients with SBO-VA may safely undergo non-operative management with close surgical monitoring (clinic follow-up and consideration of magnetic resonance enterography (MRE) to look for other aetiologies on an outpatient basis).

## Conclusions

In a nutshell, it is possible to manage SBO-VA in stable patients with conservative management first and if it fails, operative management should be considered. If the decision is to discharge a patient who has been successfully managed conservatively, close surgical follow-up should be arranged, likely a month follow-up to follow up on outpatient investigations like CT abdomen or MRI abdomen. Further research involving a larger cohort of patients is required to confidently declare that the non-operative approach is safe for stable patients who have been successfully treated with conservative approaches such as Gastrografin and nasogastric tube.
